# Niraparib activates interferon signaling and potentiates anti-PD-1 antibody efficacy in tumor models

**DOI:** 10.1038/s41598-019-38534-6

**Published:** 2019-02-12

**Authors:** Zebin Wang, Kaiming Sun, Yonghong Xiao, Bin Feng, Keith Mikule, XiaoYan Ma, Ningping Feng, Christopher P. Vellano, Lorenzo Federico, Joseph R. Marszalek, Gordon B. Mills, Jeffrey Hanke, Sridhar Ramaswamy, Jing Wang

**Affiliations:** 1 TESARO, Inc, Waltham, MA 02451 USA; 2 Center for Co-Clinical Trials, The University of Texas MD Anderson Cancer Center, Houston, TX 77030 USA; 3 Department of Systems Biology, The University of Texas MD Anderson Cancer Center, Houston, TX 77030 USA; 4 Institute for Applied Cancer Science, The University of Texas MD Anderson Cancer Center, Houston, TX 77030 USA

## Abstract

PARP inhibitors have been proven clinically efficacious in platinum-responsive ovarian cancer regardless of *BRCA1/2* status and in breast cancers with germline *BRCA1/2* mutation. However, resistance to PARP inhibitors may preexist or evolve during treatment in many cancer types and may be overcome by combining PARP inhibitors with other therapies, such as immune checkpoint inhibitors, which confer durable responses and are rapidly becoming the standard of care for multiple tumor types. This study investigated the therapeutic potential of combining niraparib, a highly selective PARP1/2 inhibitor, with anti-PD-1 immune checkpoint inhibitors in preclinical tumor models. Our results indicate that niraparib treatment increases the activity of the type I (alpha) and type II (gamma) interferon pathways and enhances the infiltration of CD8^+^ cells and CD4^+^ cells in tumors. When coadministered in immunocompetent models, the combination of niraparib and anti-PD-1 demonstrated synergistic antitumor activities in both *BRCA-*proficient and *BRCA*-deficient tumors. Interestingly, mice with tumors cured by niraparib monotherapy completely rejected tumor growth upon rechallenge with the same tumor cell line, suggesting the potential establishment of immune memory in animals treated with niraparib monotherapy. Taken together, our findings uncovered immunomodulatory effects of niraparib that may sensitize tumors to immune checkpoint blockade therapies.

## Introduction

The poly(ADP-ribose) polymerase (PARP) family of enzymes catalyzes an essential posttranslational modification process known as PARylation^1^. PARP inhibition functions by compromising the ability of tumor cells to repair DNA single-strand breaks, resulting in the accumulation of double-strand breaks (DSBs), which lead to genomic instability and, ultimately, cell death in tumor cells with homologous recombination repair deficiency^2–4^. PARP inhibition also traps PARP1/2 on DNA, forming PARP-DNA complexes that further exacerbate DNA replication fork damage. PARP inhibitors have significantly improved the clinical outcomes of ovarian and breast cancer patients, resulting in US Food and Drug Administration (FDA) approvals for the treatment of these diseases^5–7^. In platinum-responsive, high-grade serous ovarian cancers, PARP inhibitors significantly prolong progression-free survival after platinum-based chemotherapy in all patients regardless of *BRCA* status, with the highest magnitude of benefit observed in *BRCA* mutant patients^5–7^. In breast cancers, clinical response to PARP inhibitors was demonstrated in germline *BRCA* mutant patients with advanced localized or metastatic disease^8^. Despite the impressive responses seen in the clinic, the utility of PARP inhibitors as monotherapy is still limited by major challenges, such as intrinsic and acquired resistance. Therefore, combination therapy is a logical next step to broaden the patient population and confer more durable responses to PARP inhibitors.

Therapeutic antibodies against immune checkpoint proteins such as anticytotoxic T lymphocyte–associated antigen 4 (CTLA-4), anti-programmed cell death 1 (PD-1), or anti-programmed death ligand-1 (PD-L1) have emerged as promising therapies for several types of cancers, including melanoma, non-small cell lung cancer (NSCLC), renal cancer, endometrial cancer, and other cancers^9–11^. By unleashing antitumor immune responses, checkpoint inhibitors targeting inhibitory immune receptors are capable of inducing unprecedented durable responses and, in some cases, complete regression in tumors, with manageable side effects^11–13^. Nevertheless, the clinical benefits observed to date are heterogeneous and are limited to certain tumor types (e.g., melanoma and NSCLC) and patient populations (e.g., MSI-high)^11,14^. Furthermore, a substantial portion of patients, even those with sensitive tumor types such as melanoma and NSCLC, do not respond to immunotherapy^11^. To extend durable responses to more disease types and larger patient populations, there is a pressing need to establish checkpoint inhibitor-based combination strategies, such as combination with therapeutic agents capable of establishing favorable tumor immune microenvironments. For example, promising activity has been seen in the clinic when anti-PD-1/PD-L1 agents are combined with chemotherapy, which may potentially modify the tumor microenvironment^15^. In addition to direct cytotoxic effects, chemotherapeutic agents are believed to promote inflammatory tumor microenvironments and increase tumor immunogenicity^16^.

Beyond their role in inducing tumor cell death, PARP inhibitors have been shown in recent work to have potential to modulate the tumor immune microenvironment. In a *BRCA1-*deficient ovarian syngeneic model, the PARP inhibitor talazoparib induced antitumor immune effects by increasing the number of peritoneal CD8^+^ T cells and natural killer cells^17^. In breast cancer cell lines and xenograft models, PARP inhibition has been shown to upregulate PD-L1 expression in a tumor-intrinsic manner regardless of *BRCA* status^18^. In addition, both studies also showed an enhanced antitumor effect *in vivo* when PARP inhibition was combined with checkpoint blockade. However, the potential benefit of combining niraparib with a PD-1 inhibitor and the corresponding mechanism of action have not been systematically evaluated.

In this study, we investigated the effects of niraparib treatment on the tumor microenvironment and assessed the combination benefit of niraparib and anti-PD-1 therapy in *BRCA*-deficient and -proficient tumor models. Our results revealed that niraparib induced type I and type II interferon pathway activation and enhanced T cell infiltration in tumors. More importantly, synergistic antitumor activity was observed when niraparib was combined with anti-PD-1 therapy in multiple preclinical tumor models regardless of *BRCA* status. Interestingly, tumor rejection after complete regression was observed in a niraparib-sensitive model, suggesting the potential establishment of immune memory by niraparib monotherapy. Together, these data support the clinical exploration of this combination in patients.

## Materials and Methods

### RNAseq sample preparation, data generation, and processing

Frozen tumor samples were collected from *in vivo* studies in the SK6005 and MDA-MB-436 models. Total RNA was extracted and treated with DNase I to degrade any possible contaminating DNA. The mRNA was then enriched by using oligo (dT) magnetic beads. The mRNA was mixed with the fragmentation buffer and cleaved into short fragments. The first strand of cDNA was synthesized using random hexamer primers. Buffer, dNTPs, RNase H, and DNA polymerase I were added to the reaction to synthesize the second strand. The double-stranded cDNA was purified with magnetic beads, followed by end repair and 3′-end single nucleotide A (adenine) addition. Finally, sequencing adaptors were ligated to the fragments, and the fragments were enriched by PCR amplification. During the quality control step, an Agilent 2100 bioanalyzer (Agilent, Santa Clara, CA) and an ABI StepOnePlus™ Real-Time PCR system (Thermo Fisher Scientific, Waltham, MA) were used to quantify the sample library, at which point the library products were ready for sequencing via an Illumina HiSeq^TM^ 2000 (Illumina, San Diego, CA). At least 20 million clean reads were generated for each sample. After filtering, the clean reads were mapped to the reference genome using the HISAT/Bowtie2 tool. The RSEM algorithm was used to estimate the abundance of the expressed genes, and the FPKM (fragments per kilobase of transcript per million mapped reads) value was calculated for every gene^19^.

Total RNA was extracted from the FFPE samples collected from the PDX *in vivo* study using a QIAGEN RNeasy FFPE Mini Kit (cat # 74IO4) (QIAGEN, Hilden, Germany). The quantity and quality of the FFPE RNA were checked using a Nanodrop™ spectrophotometer (Thermo Fisher Scientific, Waltham, MA), a Qubit 2.0 fluorometer (Thermo Fisher Scientific, Waltham, MA), and an Agilent TapeStation 2200 (Agilent, Santa Clara, CA). cDNA libraries were constructed using TruSeq© RNA Access to capture the human exome (Illumina, San Diego, CA) according to the manufacturer’s recommendations. The cDNAs in the libraries were denatured and loaded on a NextSeq. 500 (Illumina, San Diego, CA) with v2 chemistry and run at 2 × 75 base pair read length with a mean of 25 million reads per sample. The files from each sequencing run containing the base calls per cycle were converted to FASTQ format using bcl2fastq2 conversion software (Illumina, San Diego, CA) and aligned to the Ensembl GRCh37 *Homo sapiens* reference genome using TopHat2. The aligned BAM file was then sorted using SAMtools, and the FPKM was calculated using cufflinks.

### Differential gene expression and pathway analysis

Sample gene expression FPKM values were used for 2-group paired (for PDX tumors) or unpaired (for SK6005 and MDA-MB-436 tumors) *t* tests. Differentially expressed genes (unadjusted *P* < 0.05, fold change >1.5) were selected for hypergeometric statistics based on gene set investigation (http://software.broadinstitute.org/gsea/msigdb/annotate.jsp). In addition, FPKM expression values for all genes (without selection for differential expression) were subjected to gene set enrichment analysis (GSEA; http://software.broadinstitute.org/gsea/index.jsp). Due to the small number of samples, the GSEA analysis was conducted with permutation by gene set rather than by sample.

### Immunohistochemistry

SK6005 skin tumor tissues were fixed and embedded in paraffin, and 4 µm sections were prepared for staining. Immunohistochemical staining was performed with primary antibodies specific for CD4 (Sino Bio 50134-R001), CD8 (Affymetrix 14–0808), and FoxP3 (Novus NB100–39002) on the Bond RX system (Leica Biosystems, Germany). Briefly, the sections were processed by the following incubation steps: Bond dewax solution (Leica AR9222), 0.5 min at 72 °C; Bond epitope retrieval solution 1 (Leica AR9961) or Bond epitope retrieval solution 2 (Leica AR9640), 20 minutes at 100 °C; Bond wash buffer (Leica AR9590), 3 minutes at room temperature (RT); peroxide block (Leica DS9800), 10 minutes at RT; goat serum, 20 minutes at RT for the anti-CD8 antibody; primary antibody, 60 minutes at RT; Bond wash buffer, 3 times for 2 minutes each at RT; polymer, 20–30 minutes at RT; Bond wash buffer, 3 times for 2 minutes each at RT; DAB (Leica DS9800), 5 minutes at RT; and hematoxylin (Leica DS9800), 10 minutes at RT. Five fields in each stained sample without necrosis were randomly selected and imaged at 20× magnification. All images were analyzed with ImageJ software. Positive cells were counted, and the average number of positive cells in 5 fields was taken as the score value of each sample.

### *In vivo* animal models

The protocols involving the care and use of animals were reviewed and approved by the Institutional Animal Care and Use Committees (IACUC). The care and use of animals were in accordance with the regulations of the Association for Assessment and Accreditation of Laboratory Animal Care (AAALAC).

MDA-MB-436 is a *BRCA1* mutant triple-negative breast cancer cell line known to be sensitive to PARP inhibition^20^. For the humanized MDA-MB-436 tumor model, 5 × 10^6^ MDA-MB-436 cells were inoculated subcutaneously with 50% Matrigel into the flank of 6- to 8-week-old female huNOG-EXL mice (Taconic Biosciences, US). The NOG-EXL strain of mice combines the background of severe immunodeficient CIEA NOG mice with that of human IL-3/GM-CSF-transgenic NOG mice^21^. In this study, the NOG-EXL mice were humanized by the engraftment of CD34^+^ human hematopoietic stem cells, which resulted in functional human immune cells, including B cells, T cells, macrophages, granulocytes, and monocytes, in the host mice^21^. Treatment was started when the tumors attained an average volume of 90–110 mm^3^ per group. The mice were administered 35 mg/kg niraparib orally once daily for 5 days on and 2 days off per week, pembrolizumab (200 mg daily dosing twice weekly) and the combination for 28 days. For the nonhumanized MDA-MB-436 model, 5 × 10^6^ MDA-MB-436 cells were inoculated subcutaneously with 50% Matrigel into the flank of 6- to 8-week-old female NOG mice (Taconic Biosciences, US). Treatment was started when the tumors attained an average volume of 80–120 mm^3^ per group. The mice were administered 50 mg/kg niraparib orally once daily for 27 days.

SK6005 (Crown Bioscience, China) is a mouse skin tumor model established from a C57BL/6J-Apc^Min^ heterozygous mouse that does not carry deleterious *BRCA1/2* mutations. C57BL/6 mice were inoculated subcutaneously in the right flank with a primary SK6005 tumor fragment (2–4 mm in diameter) for tumor development. The animals were randomized when the tumors attained an average volume of approximately 140 mm^3^. The mice were administered 50 mg/kg or 25 mg/kg niraparib orally once daily, anti-PD-1 antibody (BioXCell RMP1–14), or the combination.

For the *BRCA1*-deficient ovarian carcinoma tumor model (BRKras), 10 × 10^6^ *BRCA1*-null ovarian cancer cells were inoculated subcutaneously into the flank of 7- to 8-week-old female FVB mice (Charles River Laboratories, Wilmington, MA). Treatment was started when the tumors attained an average volume of 90 mm^3^ per group, on day 9 after tumor inoculation. Mice were treated with 30 mg/kg or 50 mg/kg niraparib orally once daily, anti-PD-1 antibody (BioXCell RMP1–14), or the combination between day 9 and day 29 for 21 days. Tumor growth was monitored twice weekly. On day 29, the treatments were stopped, and tumor growth was monitored from day 29 to day 64. For the rechallenge experiment, 10 × 10^6^ *BRCA1*-deficient ovarian cancer cells were inoculated subcutaneously into the other flank of the same mice. Tumor growth was monitored for an additional 5 weeks from day 65 post inoculation.

For the syngeneic MMTV-LPA1-T22 model, female 6- to 8-week-old FVB mice (Jackson Lab, Bar Harbor, ME) were implanted in the right side fourth fat pad with a 3 × 3 mm LPA1-T22 tumor fragment. When the tumors attained an average volume of 100–150 mm^3^, the mice were randomized, and dosing was initiated (day 0). The mice were treated with control, niraparib, anti-PD-1 (2C4 mouse surrogate for TSR-042), or the combination of niraparib + anti-PD-1 for 16 days. The niraparib dosage was 50 mg/kg orally once daily, and the anti-PD-1 antibody dosage was 10 mg/kg twice weekly, both as monotherapy and in combination. SA9003 (Crown Bioscience, China) is a mouse syngeneic sarcoma transplant tumor model established from a spontaneous sarcoma developed from *TP53*−/− C57BL/6 mice. C57BL/6 mice were inoculated subcutaneously in the right flank with a primary SA9003 tumor fragment (2–4 mm in diameter) for tumor development. When the tumors attained an average volume of 80–100 mm^3^, the mice were randomized, and dosing was initiated (day 0). The mice were administered control, niraparib, anti-PD-1 (BioXCell RMP1–14), or the combination of niraparib + anti-PD-1 for 16 days. at the niraparib dosage was 50 mg/kg orally once daily, and the anti-PD-1 antibody dosage was 10 mg/kg twice weekly, both as monotherapy and in combination. The KLN205 murine lung squamous syngeneic tumor model was established by injecting KLN205 cells subcutaneously into syngeneic DBA/2 mice. When the tumors attained an average volume of 100–150 mm^3^, the mice were randomized, and dosing was initiated (day 0). The mice were administered control, niraparib, anti-PD-1 (2C4 mouse surrogate for TSR-042), or the combination of niraparib + anti-PD-1. at the niraparib dosage was 50 mg/kg orally once daily for 22 days, and the anti-PD-1 antibody dosage was 5 mg/kg twice weekly for two weeks. The MC38 murine colon syngeneic model was established by subcutaneously injecting MC38 cells into syngeneic C57BL/6 mice. When the tumors attained an average volume of 50–100 mm^3^, the mice were randomized, and dosing was initiated (day 0). The mice were administered control, niraparib, anti-PD-1 (BioXCell RMP1–14), or the combination of niraparib + anti-PD-1. at the niraparib dosage was 50 mg/kg orally once daily, and the anti-PD-1 antibody dosage was 0.5 mg/kg twice weekly for the first two weeks. BL6078 (Crown Bioscience, China) is a mouse syngeneic bladder transplant tumor model established from the bladder tumor of a C57BL/6 mouse resulting from the conditional activation of the KrasG12D mutation and PTEN deletion in the bladder. C57BL/6 mice were inoculated subcutaneously in the right flank with a primary BL6078 tumor fragment (2–4 mm in diameter) for tumor development. When the tumor attained an average volume of 100–150 mm^3^, the mice were randomized, and dosing was initiated (day 0). The mice were administered control, niraparib, anti-PD-L1 (BioXCell 10 f.9G2), or the combination of niraparib + anti-PD-L1 for 19 days. The niraparib dosage was 50 mg/kg orally once daily, and the anti-PD-1 antibody dosage was 10 mg/kg twice weekly, both as monotherapy and in combination. The dose is expressed in mg/kg, which indicates the amount of drug (in milligrams) per kilogram of body weight. For all *in vivo* models, tumor growth was monitored twice weekly by caliper measurement. Tumor volume was calculated using the following formula: tumor volume = 0.5 × long diameter × short diameter^2^. Niraparib tosylate was formulated in methyl cellulose, and anti-PD-1 antibodies were diluted in phosphate-buffered saline.

### Flow cytometry

MDA-MB-436 tumors were collected from huNOG-EXL mice and processed with a Miltenyi MACS Tumor Dissociation Kit on a gentleMACS dissociator (Miltenyi Biotec, Germany), following the manufacturer’s protocol. Single-cell suspensions were labeled with the following antibodies: anti-CD45-eFluor 506 (eBioscience 69–0459–42); anti-CD3-eVolve 655 (eBioscience 86-0037-42); anti-CD4-FITC (eBioscience 11-0049-42); anti-CD8-PE-Cy7 (eBioscience 25-0088-42); anti-FoxP3-eFluor 450 (eBioscience 48-4776-42); anti-Ki67-PerCP-eFluor710 (eBioscience 46-5699-42); and viability dye (eBioscience 65-0865-18). Flow cytometry was performed on an Attune NxT flow cytometer (Thermo Fisher, Waltham, US).

### Immunoblots

Cell lysates were prepared using 1 × RIPA buffer (EMD Millipore) consisting of 50 mM Tris-HCl, pH 7.4; 150 mM NaCl; 0.25% deoxycholic acid; 1% NP-40; 1 mM EDTA; and freshly added Halt™ Protease and Phosphatase Inhibitor Single-Use Cocktail (Thermo Fisher, Waltham, US). Protein concentrations were measured using a BCA protein assay kit (Thermo Fisher, Waltham, US). The following antibodies were used: anti-STING-pS366 (CST 85735); anti-STING (CST 13647); anti-TBK1-pS172 (CST 5483); anti-TBK1 (CST 3504); anti-NFκb p65-pS536 (CST 3033); anti-NFκb p65 (CST 8242); and anti-GAPDH (Biolegend HRP Anti-GAPDH Ab 649203). Horseradish peroxidase-conjugated secondary antibodies (CST) were used to amplify the signal from the primary antibodies.

### RT-qPCR analysis

MDA-MB-436 and DLD1 BRCA2−/− cells were lysed in RLT buffer (Qiagen #79216) containing 1% β-mercaptoethanol, followed by mRNA purification using Promega’s SV96 Total RNA Isolation System (Z3500) according to the manufacturer’s instructions (Promega, Madison, WI). The expression of IFNB1 (TaqMan assay ID Hs01077958_s1) and IFNA1 (TaqMan assay ID Hs04189288_g1) was then determined by RT-qPCR (a Life Technologies TaqMan RNA-to-CT 1-step kit) on a QS6 thermal cycler (Thermo Fisher, Waltham, US). The relative quantification was determined using the ΔΔCt method, where a duplexed control (HPRT1, TaqMan assay ID Hs02800695_m1) was included, and expression changes were normalized to the expression in the respective reference sample (DMSO), which was set to 1 (log2(1) = 0).

### Statistical analysis

Statistical significance was calculated by Student’s *t* test using GraphPad Prism 7.0. Statistically significant changes are indicated with asterisks. 

## Results

### Niraparib treatment induced the activation of type I and type II interferon pathways in both immunocompetent *BRCA*-proficient and *BRCA*-deficient tumors

**Figure 1:**
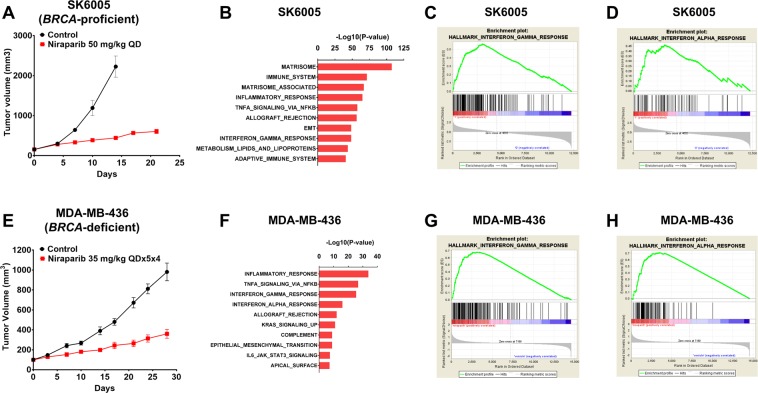
Interferon response signature genes were significantly enriched in niraparib-treated tumors. (**A**) Tumor growth curve for the SK6005 syngeneic model with control or 50 mg/kg niraparib (QD) treatment. (**B**) Genes identified with DEseq as significantly upregulated upon niraparib treatment (p <=0.05, fold change >=1.5) were subjected to enrichment analysis of pathway gene sets. Gene Set Enrichment Analysis (GSEA) demonstrated a significant enrichment of interferon gamma signature (**C**) and interferon alpha signature (**D**) genes in niraparib-treated samples. **(E)** Tumor growth curve for the MDA-MB-436 NOG-EXL humanized model treated with control or 35 mg/kg niraparib daily for 5 days on and 2 days off for 4 weeks (QD × 5 × 4). (**F**) Significantly upregulated genes identified with a two-sample *t* test (p <=0.05, fold change >=1.5) were subjected to enrichment analysis of pathway gene sets. Gene Set Enrichment Analysis (GSEA) demonstrated a significant enrichment of interferon gamma signature (**G**) and interferon alpha signature (**H**) genes in niraparib-treated samples.

In addition to the tumor-intrinsic cytotoxicity of PARP inhibitors attributed to synthetic lethality with homologous recombination repair deficiency and PARP trapping^2–4,22^, PARP inhibition has been suggested by emerging evidence to modify the immune context of tumors^11,17,18,23^. To investigate the potential immunomodulatory effect of niraparib and its molecular basis, a functional genomics approach was utilized to evaluate tumoral gene expression changes induced by niraparib treatment *in vivo*. Tumor samples were collected from two niraparib-responsive immunocompetent tumor models, the *BRCA-*proficient SK6005 mouse syngeneic model (Fig. 1A) and the humanized NOG-EXL *BRCA*-deficient MDA-MB-436 triple-negative breast cancer xenograft model (Fig. 1E), at the end of the efficacy studies. Pathway enrichment analysis of differentially expressed genes identified by RNA sequencing revealed that immune-related gene expression signatures, such as the inflammatory response, TNFA signaling response, interferon gamma response, and interferon alpha response signatures, demonstrated the highest correlation with niraparib treatment in both tumor models regardless of *BRCA* mutation status (Fig. 1B,F). Consistent with these results, the independent Gene Set Enrichment Analysis (GSEA) also identified significant activation of hallmark interferon alpha and gamma response genes upon niraparib treatment in both *BRCA*-proficient and *BRCA*-deficient models (Fig. 1C,D,G,H). Both the interferon alpha and gamma signatures consist of genes whose expression is known to be upregulated in response to alpha or gamma interferon according to the Molecular Signatures Database (MSigDB 6.1)^24,25^. These results demonstrated that niraparib treatment activates interferon signaling in both *BRCA*-proficient and *BRCA*-deficient tumors established in immunocompetent mouse models.

### Niraparib promoted tumor immune cell infiltration in both *BRCA*-proficient and *BRCA*-deficient tumor models

**Figure 2:**
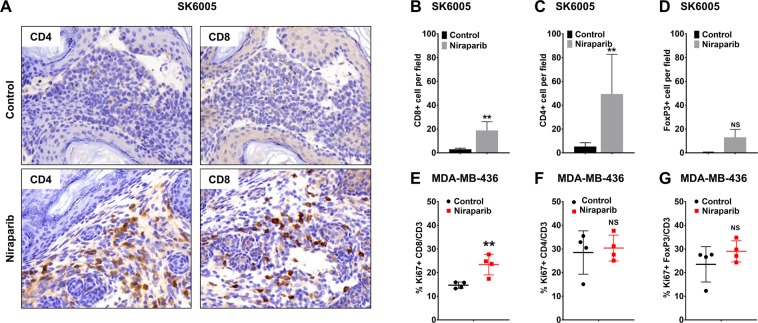
Niraparib promoted tumor immune cell infiltration in both the *BRCA*-proficient SK6005 syngeneic and *BRCA*-deficient MDA-MB-436 NOG-EXL humanized tumor models (**A**) Representative images of CD4 and CD8 immunohistochemical staining in control- and niraparib-treated *BRCA*-proficient SK6005 tumors. (**B**–**D**) Quantification of the number of CD4^+^ cells, CD8^+^ cells and FoxP3^+^ cells per field upon niraparib treatment in *BRCA*-proficient SK6005 tumors. (**E-G**) Percentage of Ki67-positive CD4^+^ cells, CD8^+^ cells and FoxP3^+^ cells among the total CD3^+^ population by flow cytometry in humanized NOG-EXL MDA-MB-436 tumors. **p-value is less than 0.05 by Student’s *t* test.

Because the activation of interferon signaling pathways has a pivotal role in modulating the tumor microenvironment via effects such as the augmentation of immune cell activity^26,27^, we next evaluated the immune cell composition of niraparib-responsive tumors following repeated daily dosing with niraparib. In the niraparib-sensitive (Fig. 1A) *BRCA*-proficient SK6005 mouse syngeneic transplant model, immunohistochemical staining of immune cell surface markers revealed profound increases in the number of CD4^+^ and CD8^+^ cells upon niraparib treatment (Fig. 2A–C), suggesting that niraparib significantly increased the infiltration of CD4^+^ and CD8^+^ immune cells. A nonsignificant trend of FoxP3^+^ cell induction was also observed upon niraparib treatment (Fig. 2D), suggesting the promotion of overall immune cell infiltration, including regulatory T cell infiltration, in this model.

The immunomodulatory effects of niraparib in the human tumor microenvironment were further evaluated in the niraparib-sensitive (Fig. 1E) *BRCA1* mutant MDA-MB-436 TNBC tumors established in humanized NOG-EXL mice. Multicolor flow cytometry analysis revealed an increase in proliferating CD8^+^ T cells after 2 weeks of niraparib treatment at 35 mg/kg on a 5 day on/2 day off schedule (Fig. 2E). In comparison, proliferating CD4^+^ T cells (Fig. 2F) and proliferating FoxP3^+^ T cells (Fig. 2G) did not increase significantly, suggesting that the increase is specific to proliferating CD8^+^ T cells in MDA-MB-436 tumors. Overall, these results indicate that niraparib modified the tumor immune microenvironment by increasing the intratumoral immune cell numbers in both *BRCA*-proficient and *BRCA*-deficient tumors.

### Niraparib-induced interferon activation is present both in xenograft tumors established in immunocompromised mice and in cultured tumor cells

**Figure 3:**
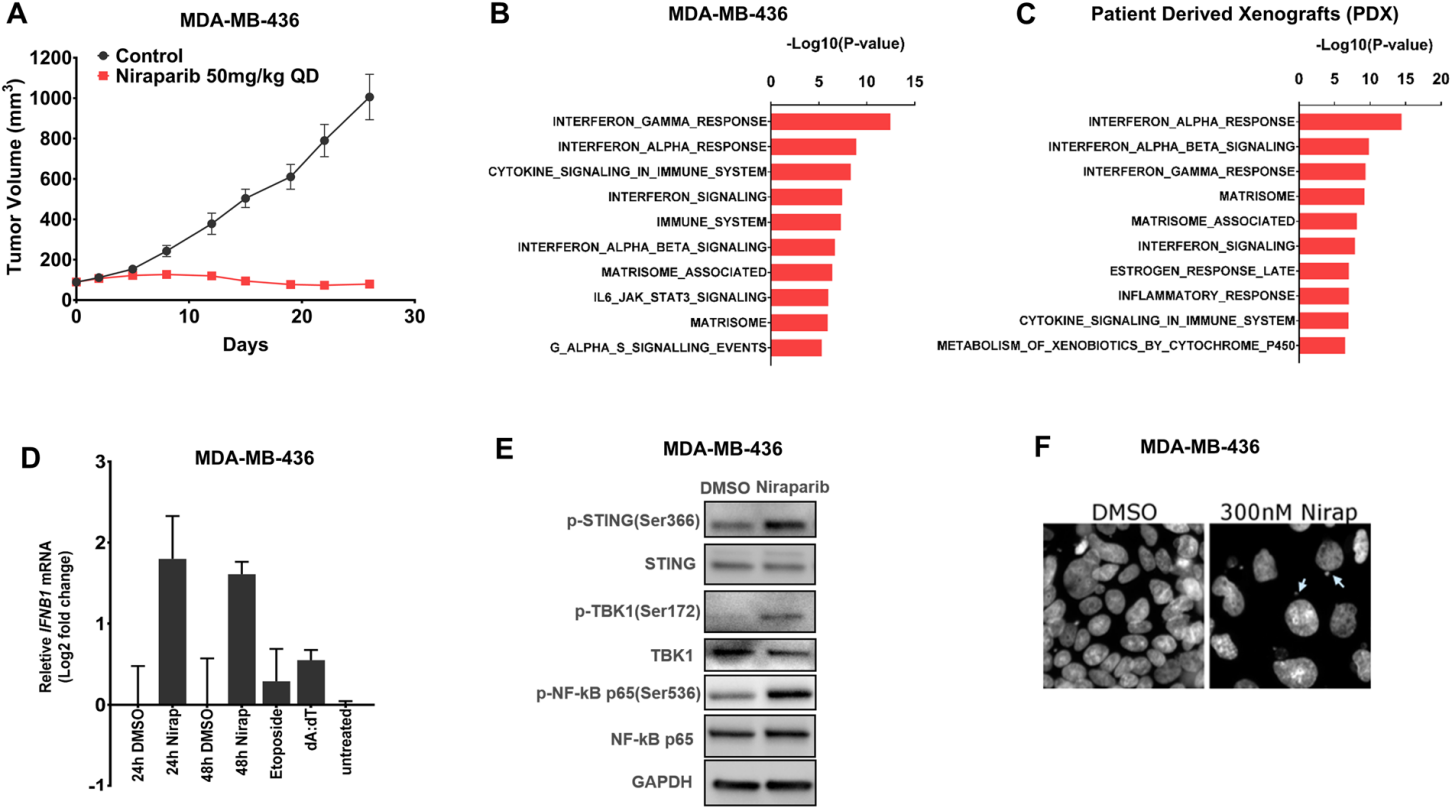
Niraparib-induced interferon activation is present in xenograft tumors established in immunocompromised mice and in cultured tumor cells (**A**) Tumor growth curve for MDA-MB-436 tumors in an immunodeficient NOG model treated with control or 50 mg/kg niraparib (QD). **(B)** Genes identified with a two-sample t-test as significantly upregulated upon niraparib treatment (p <= 0.05, fold change >=1.5) in MDA-MB-436 tumors in immune-deficient NOG mice were subjected to enrichment analysis of pathway gene sets. **(C)** Genes identified with a paired two-sample *t* test as significantly upregulated upon niraparib treatment (p <=0.05, fold change >=1.5) in 8 niraparib sensitive PDX models were subjected to enrichment analysis of pathway gene sets. **(D)** mRNA expression of *IFNB1* upon niraparib treatment (300 nM, 24 h and 48 h), etoposide treatment (50 µM, 18 h), or dA:dT transfection (0.5 µg/ml) in MDA-MB-436 cells. **(E)** Protein expression of p-STING (Ser366), STING, p-TBK1 (Ser172), TBK1, p-NF-κB p65 (Ser536) and NF-κB p65 upon 1 µM niraparib treatment (48 h) by western blotting from the same MDA-MB-436 lysate run on different gels indicated by the divider lines. (**F**) DAPI staining of nuclear structures following DMSO or 300 nM niraparib treatment in MDA-MB-436 cells cultured *in vitro* with arrows indicating micronuclei formation in niraparib-treated cells *in vitro*.

Because both tumor and immune cells could contribute to interferon pathway activation in response to interferon stimulation^27^, we next analyzed the tumor-specific transcriptome changes in niraparib-sensitive MDA-MB-436 tumors developed in immunodeficient host mice (Fig. 3A) to determine the origin of the niraparib-induced interferon pathway activation. Pathway enrichment analysis of the differentially expressed genes revealed increased activities of type I and type II interferon pathways upon niraparib treatment in MDA-MB-436 xenograft tumors (Fig. 3B). In addition, RNA profiling analysis using the same approach was performed in 8 niraparib-sensitive patient-derived xenograft (PDX) models derived from breast, lung, and bladder tumors and established in immunocompromised mice (Supplemental Fig. 1). Pathway enrichment analysis of the differentially expressed genes again revealed increased activities of type I and type II interferon pathways upon niraparib treatment in these PDX tumors established in immunocompromised mice (Fig. 3C). Consistent with this result, increased expression of chemotherapy-induced type I interferon-stimulated genes, such as RSAD2, OAS2, MX2, IFIT2, and MX1^28^, was also observed in these PDX models (Supplemental Fig. 2A–C). These results suggest that niraparib-induced activation of the type I interferon pathway may not require the presence of a competent immune system.

Because the immune-deficient models may still contain residual mouse immune system, the expression of type I interferon RNA following niraparib treatment was assessed in cultured MDA-MB-436 cells, to further explore the origin of niraparib-induced type I interferon pathway activation. Although *IFNA1* (interferon alpha) expression was not detectable (data not shown), niraparib treatment for 24 and 48 hours resulted in a 3- to 4-fold increase in the mRNA expression of *IFNB1* (interferon beta) (Fig. 3D) in MDA-MB-436 cells *in vitro*. Consistent with these results, niraparib also increased the *IFNB1* mRNA levels in DLD1 *BRCA2*−/− cells (Supplemental Fig. 2E). In contrast to its undetectability in MDA-MB-436 cells, *IFNA1* mRNA was detectable in DLD1 *BRCA2*−/− cells cultured *in vitro*, and *IFNA1* mRNA expression was induced upon niraparib treatment (Supplemental Fig. 2D). Given that tumor cells are the only cells present in the *in vitro* culture system, these results clearly indicate that niraparib treatment induced the mRNA expression of type I interferon genes in a tumor-intrinsic manner.

Because the activation of the cGAS/STING pathway has been well documented as a tumor cell-intrinsic mechanism mediating the DNA damage-induced proinflammatory immune response^29,30^, STING pathway activity was next evaluated in MDA-MB-436 cells. Our results showed that in cultured MDA-MB-436 cells, niraparib treatment at 1 µM for 48 hours led to elevation of p-TBK1 (Ser172) and p-NF-κB p65 (Ser536), which have been demonstrated to induce the expression of immunomodulatory cytokines, including type I interferons^31^ (Fig. 3E). STING phosphorylation at Ser366, a critical event for STING downstream signaling and immune activation^32,33^, was also observed under the same conditions (Fig. 3E). The increased phosphorylation of STING was also observed in *BRCA*wt TNBC MDA-MB-231 cells, when treated with niraparib (Supplemental Fig. 3). In addition, micronuclei formation, which has been recently discovered to be associated with STING activation in tumor cells^7,31,34–37^, was observed upon niraparib treatment in both MDA-MB-436 cells (Fig. 3F) and DLD1 *BRCA−/−* cells (Supplemental Fig. 2F) *in vitro*. Collectively, these results suggest that niraparib induced cGAS/STING activation in tumor cells, which may play a role in tumor-intrinsic type I interferon induction.

### Combination therapy with niraparib and anti-PD-1 augmented antitumor activity and conferred durable responses in *BRCA*-deficient tumor models

**Figure 4:**
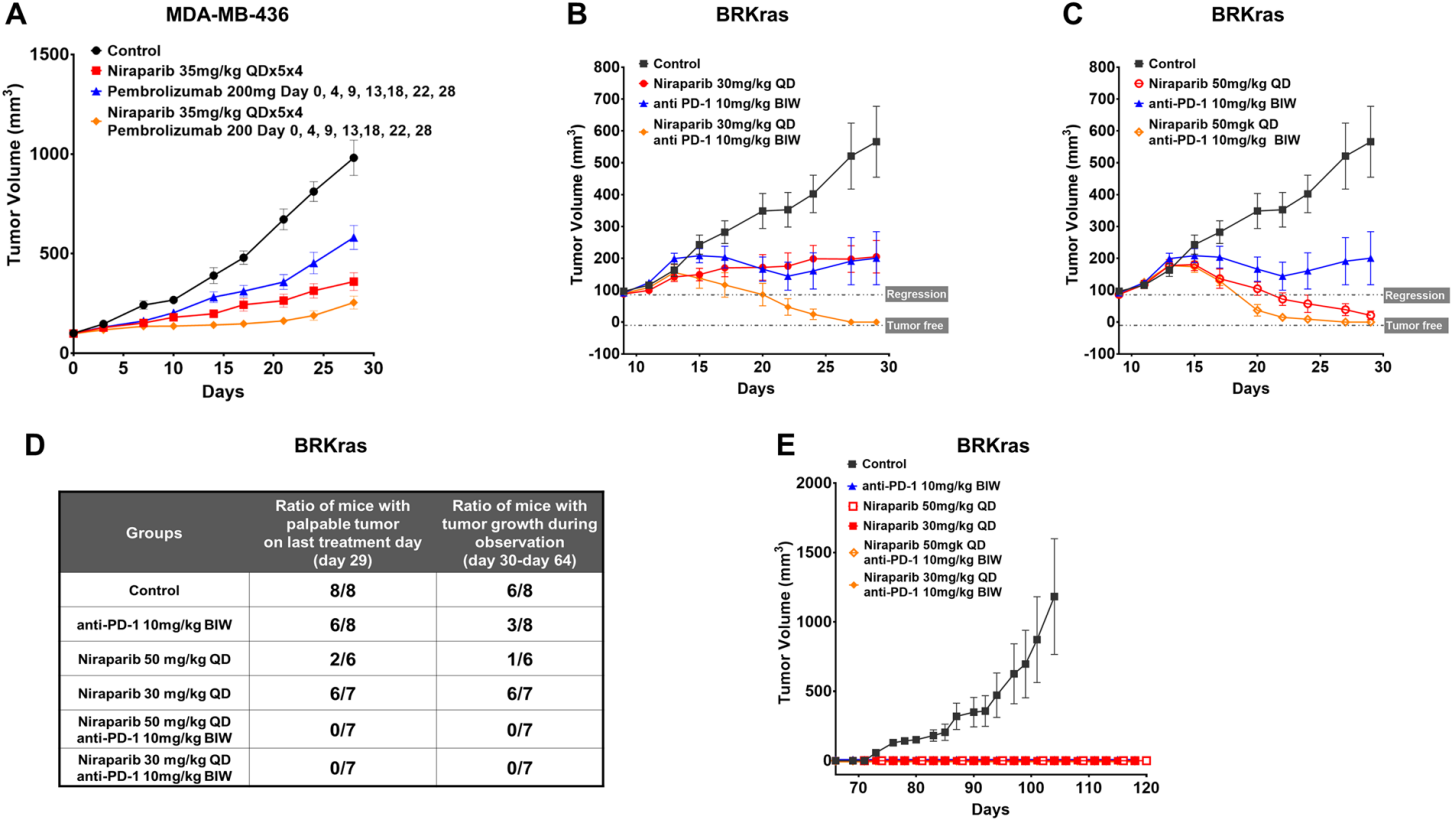
Combination therapy with niraparib and anti-PD-1 augmented antitumor activity and conferred durable responses in *BRCA*-deficient tumor models (**A**) Tumor growth in the *BRCA-*deficient MDA-MB-436 model in huNOG-EXL mice treated with 200 mg anti-PD-1 (pembrolizumab) on days 0, 4, 9, 13, 18, 22, and 28; 35 mg/kg niraparib daily for 5 days on and 2 days off for 4 weeks; and the combination of these agents. (**B**) The *BRCA*1-null ovarian cancer mouse syngeneic model was treated with 30 mg/kg niraparib QD, 10 mg/kg anti-PD-1 (BioXCell RMP1-14) BIW, and the combination of these agents for 21 days (day 9–29). Tumor regrowth was monitored post treatment (days 29–64). (**C**) The *BRCA*1-null ovarian cancer mouse syngeneic model was treated with niraparib 50 mg/kg QD, anti-PD-1 10 mg/kg BIW, and the combination of these agents for 21 days (days 9–29). Tumor regrowth was monitored post treatment (days 29–64). **(D)** Table summarizing the ratio of mice with palpable tumors on day 29 (last treatment day) and the ratio of mice with tumor growth observed during the drug-free, posttreatment observation period (days 30–64). **(E)** Growth curves for rechallenge with *BRCA1*-null ovarian cancer cells implanted on day 65 in the tumor-free mice from (**D**) and age-matched treatment-naïve mice.

Given the observation that niraparib modulates the tumor immune microenvironment to provide favorable conditions for immune checkpoint therapies, we next evaluated the potential therapeutic benefit of combining niraparib with anti-PD-1 therapies in immunocompetent mouse models. In the humanized NOG-EXL *BRCA*-deficient MDA-MB-436 triple-negative breast cancer xenograft model, the combination of niraparib (35 mg/kg daily dosing on a 5 day on/2 day off schedule) and pembrolizumab (200 mg daily dosing twice weekly) demonstrated significantly better antitumor effects, with 75% tumor growth inhibition (TGI) than either niraparib (TGI = 63% p = 0.0384) or pembrolizumab (TGI = 41% p = 0.0140) monotherapy after 4 weeks of treatment (Fig. 4A), suggesting a combination benefit in this model. The combination of niraparib and a mouse PD-1 blocking antibody was next evaluated in a *BRCA1*-deficient ovarian carcinoma syngeneic model (BRKras) previously established to resemble features of human metastatic serous ovarian cancer^38,39^, which also harbors the *KrasG12D* mutation, expresses c-Myc and Akt and is *TP53*-null. Dose-dependent responses to oral daily niraparib monotherapy were observed in this model, as the treatment of established tumors with a 50 mg/kg or 30 mg/kg daily dose of niraparib drove complete regression or 64% TGI, respectively, whereas anti-PD-1 monotherapy led to 65% TGI at the end of the study (Fig. 4B,C). The combination of a suboptimal dose (30 mg/kg) of niraparib and an anti-PD-1 antibody significantly improved the tumor responses compared with either monotherapy alone and resulted in complete tumor regression (Fig. 4B). A combination benefit was also observed when a full dose of niraparib (50 mg/kg) was combined with anti-PD-1 therapy in this model, as this combination achieved complete regression sooner than full-dose niraparib monotherapy (Fig. 4C).

To evaluate the durability of response, a treatment-free observation period was incorporated in all arms on day 29 after 3 weeks of dosing. During the 5-week observation period, the tumor regrowth rate in the 2 combination arms was markedly lower than that in the arms that received the single agent, with none of the tumors in the 2 combination arms developing any signs of tumor growth, suggesting the durable antitumor effect of the combination of niraparib and anti-PD-1 (Fig. 4D).

On study day 65, at the end of the observation period, all tumor-free mice were rechallenged by the inoculation of the same ovarian cancer cell line. Age-matched treatment-naïve mice were also inoculated with the same number of tumor cells as the treated mice. Seven weeks post reinoculation, none of the tumor-free mice from the combination and single-agent arms developed any signs of tumor growth, whereas tumors grew normally in the age-matched control mice (Fig. 4E), suggesting the potential establishment of immune memory by niraparib and its combination with anti-PD-1 therapy in *BRCA*-deficient tumors.

Collectively, these results demonstrated a combination benefit of niraparib and anti-PD-1 therapy in *BRCA*-deficient models, with rapid and durable effect and the potential establishment of immune memory.

### Combination therapy with niraparib and anti-PD-1 demonstrated a synergistic antitumor effect and combination benefit in *BRCA*-proficient tumor models

Encouraged by the combination benefit observed in the *BRCA*-deficient models, we next tested the same combination in a collection of *BRCA*-proficient syngeneic models to explore potential combination benefits in tumors with intact BRCA function. We first investigated the combination in the mouse syngeneic skin cancer model SK6005. Given that the SK6005 tumors were sensitive to niraparib treatment at 50 mg/kg, we chose a niraparib dose of 25 mg/kg to reveal the combination potential. SK6005 was not sensitive to either full-dose anti-PD-1 or low-dose niraparib, which resulted in 11% and 16% tumor growth inhibition, respectively, when used as monotherapy. Combination therapy with niraparib and anti-PD-1 resulted in tumor growth inhibition of approximately 44% in SK6005 tumors, which was greater than the combined effect of the two agents, suggesting the presence of cooperativity between niraparib and anti-PD-1 therapy.

**Figure 5:**
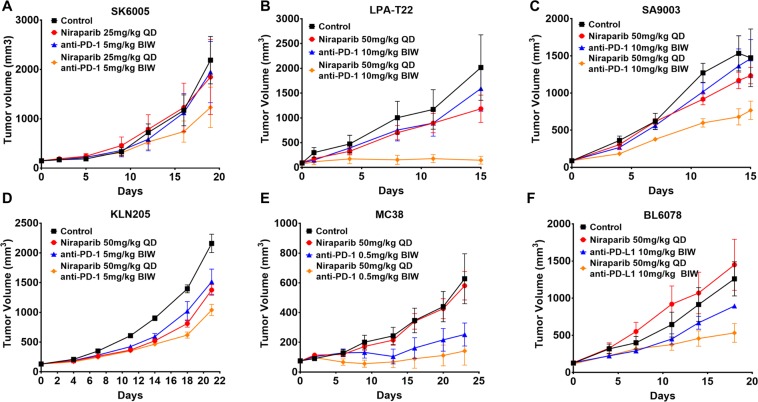
Combination therapy with niraparib and an anti-PD-(L)1 antibody demonstrated significantly enhanced antitumor activity in a *BRCA*-proficient mouse syngeneic model **(A)** Tumor growth in the SK6005 skin syngeneic transplant model treated with anti-PD-1 (BioXCell RMP1–14), niraparib, and the combination of these agents. Niraparib was administered orally at 25 mg/kg daily, and the anti-PD-1 antibody was administered intraperitoneally at 5 mg/kg twice weekly. **(B)** Tumor growth in the MMTV-LPA1-T22 syngeneic transplant models treated with anti-PD-1 (2C4), niraparib, and the combination of these agents. Niraparib was administered orally at 50 mg/kg daily, and the anti-PD-1 antibody was administered intraperitoneally at 10 mg/kg twice weekly. **(C)** Tumor growth in the SA9003 sarcoma syngeneic model treated with anti-PD-1 (BioXCell RMP1–14), niraparib, and the combination of these agents. Niraparib was administered orally at 50 mg/kg daily, and the anti-PD-1 antibody was administered intraperitoneally at 10 mg/kg twice weekly. **(D)** Tumor growth in the KLN205 lung syngeneic model treated with anti-PD-1 (2C4), niraparib, and the combination of these agents. Niraparib was administered orally at 50 mg/kg daily, and the anti-PD-1 antibody was administered intraperitoneally at 5 mg/kg twice weekly. **(E)** Tumor growth in the MC38 colon syngeneic model treated with anti-PD-1 (BioXCell RMP1–14), niraparib, and the combination of these agents. Niraparib was administered orally at 50 mg/kg daily, and the anti-PD-1 antibody was administered intraperitoneally at 0.5 mg/kg twice weekly. **(F)** Tumor growth in the BL6078 bladder syngeneic model treated with anti-PD-L1 (BioXCell 10 f.9G2), niraparib, and the combination of these agents. Niraparib was administered orally at 50 mg/kg daily, and the anti-PD-L1 antibody was administered intraperitoneally at 10 mg/kg twice weekly.

The combination of niraparib and anti-PD-1 therapy was next evaluated in the breast cancer syngeneic transplant MMTV-LPA1-T22 model established by the sequential orthotopic transplantation of tumors originating from autotaxin (ATX)-lysophosphatidic acid receptor transgenic mice^40,41^. Mice treated with either niraparib (50 mg/kg) or anti-PD-1 (10 mg/kg) monotherapy showed moderate responses, with TGI of 45% and 30%, respectively. In comparison, the combination of these two drugs at the same doses led to a significantly improved antitumor response, with 91% TGI (Fig. 5B), which was greater than the numerical sum of the TGI induced by each single agent, indicating that synergistic antitumor activity was achieved with this combination.

The antitumor effect of this combination was also evaluated in a tumor model refractory to anti-PD-1 therapy, the SA9003 syngeneic transplant sarcoma model generated on the *TP53*-null background. Combination treatment resulted in 51% TGI, whereas neither single agent demonstrated any meaningful tumor growth inhibition (Fig. 5C), suggesting a synergistic antitumor effect in this model with niraparib and PD-1 resistance.

In the KLN205 lung squamous cell carcinoma syngeneic model, the combination of niraparib and anti-PD-1 demonstrated enhanced antitumor activity and resulted in 52% TGI, compared to the results of niraparib monotherapy, with 36% TGI, and anti-PD-1 monotherapy, with 30% TGI (Fig. 5D), suggesting a combination benefit in this model.

MC38 is a murine colon adenocarcinoma syngeneic model that was reported to be responsive to anti-PD-1 monotherapy^42^. MC38 was not sensitive to niraparib monotherapy; however, the addition of niraparib improved the tumor response to anti-PD-1 therapy (Fig. 5E).

The combination benefit was not limited to the anti-PD-1 agent, as synergistic antitumor activity was observed when niraparib and anti-PD-L1 therapy were combined in the niraparib-resistant bladder syngeneic model BL6078 (Fig. 5F). The combination of niraparib and the anti-PD-L1 antibody resulted in 66% TGI, which was significantly improved compared with the antitumor effect of anti-PD-L1 monotherapy, which achieved 10% TGI (p = 0.0102); these results suggested a synergistic antitumor effect in this niraparib-resistant model. Collectively, these results demonstrated the synergistic antitumor effect or combination benefit of combination therapy with niraparib and anti-PD-(L)1 over those of the single agents in multiple *BRCA*-proficient tumor models. Interestingly, the presence of the synergistic combination benefit did not seem to be correlated with the response to either monotherapy.

Taken together, our results revealed the immunomodulatory effects of niraparib, including interferon pathway activation and immune cell infiltration enhancement, and demonstrated the durable therapeutic benefit of combination therapy with niraparib and anti-PD-1 in both *BRCA*-deficient and *BRCA*-proficient models. These findings support the development of combination therapies with niraparib and anti-PD-1 in clinical settings.

## Discussion

Our results provide new insights into the immunoregulatory functions of the PARP inhibitor niraparib and demonstrate the therapeutic potential of combining niraparib with anti-PD-1 therapy in preclinical models. These findings stemmed from the observation that significantly enriched interferon pathway signatures, including both type I and type II interferon pathway signatures, were identified in niraparib-treated tumors through genome-wide transcriptome profiling. Consistent with the transcriptome changes, a significant increase in the number of immune cells within the intratumoral compartments was also observed, indicating an active antitumor immune response upon niraparib treatment. In addition, these niraparib induced changes in tumor immune microenvironment may at least partially originate from tumor cells, based on the results from cultured cells and tumors established in immunocompromised mice. Importantly, niraparib-induced alteration of the tumor microenvironment favored its combination with anti-PD-1 therapy, in which niraparib and anti-PD-1 demonstrated synergistic antitumor activity in both *BRCA* mutant and *BRCA* wild-type tumor models. Our data uncovered the potential immunomodulatory functions of niraparib and supported the combination of niraparib with immunotherapeutic agents.

The correlated observation in cultured MDA-MB-436 cells suggested that one of the mechanisms through which niraparib may stimulate type I interferon expression in tumor cells acts through the activation of the STING pathway. Upon niraparib treatment, we observed an increase in STING phosphorylation at Ser366 and elevated phosphorylation of two key downstream effectors, p-TBK1 (Ser172) and p-NF-κB p65 (Ser356), both of which have been linked to an increase in type I interferon production^31^. The activation of the cGAS/STING pathway might be a result of the accumulation of unrepaired DNA damage in MDA-MB-436 cells upon niraparib treatment. DNA damage has been linked to the activation of antitumor immune responses via multiple mechanisms, including the STING pathway. It was reported that unrepaired DNA lesions due to ATM dysfunction in ataxia-telangiectasia patients resulted in spontaneous type I interferon (IFN) responses in humans. *ATM* deficiency led to the constant production of type I IFNs and primed innate immune mechanisms for the amplified response via the activation of the cGAS/STING pathway in immune cells^37^. In the context of human malignancies, endogenous dsDNA breaks caused by DNA-damaging agents such as etoposide or ionized irradiation could also trigger the induction of interferon-stimulated gene expression via cGAS/STING^43,44^. In addition, recent studies suggested that micronuclear DNA functions as a proinflammatory molecular entity, where micronuclei-localized cGAS/STING senses the presence of DNA damage and induces immune responses^34–36^. Similar findings on the activation of STING pathways and the induction of interferon-stimulated genes have been reported with talazoparib and rucaparib, two additional PARP inhibitors, suggesting that the immunomodulatory effects are mostly mediated by PARP1/2 inhibition instead of off-target activities. Furthermore, type II (gamma) interferon pathway was activated upon niraparib treatment together with type I (alpha) interferon pathway consistently in several models suggesting the potential elevation of IFN gamma expression within tumor and tumor-microenvironment upon niraparib treatment. The production of IFN gamma was transient and limited to certain immune cells such as T and NK cells, it is therefore challenging to monitor from *in vivo* experiments^45–47^. Type I interferon signaling could contribute to type II interferon signaling linking innate immunity to adaptive immunity functions^48^. Type I interferon production was elevated upon niraparib in tumor cells which could at least partially explain the type II interferon pathway activation upon niraparib treatment.

In this study, we uncovered a novel immunomodulatory function of niraparib via the activation of interferon pathways and observed enhanced CD4^+^ and CD8^+^ immune cell infiltration upon niraparib treatment, which accompanied the elevated interferon pathway activity. Previously, PARP inhibition by BMN 673 was shown to increase the number and function of peritoneal CD8^+^ and NK cells. However, the induction of effector cells was accompanied by an increase in FoxP3^+^ CD4^+^ Treg cells^17^. In comparison, a trend of Treg induction was observed in this study upon niraparib treatment, but this trend was not statistically significant, suggesting potential differences in immunomodulatory effects among different PARP inhibitors, which may potentially attribute to their direct impacts on immune cells or the specific tumor immune microenvironment in certain tumor models. Further research is warranted, in order to fully understand this potential differentiation. In addition, our results demonstrated that STING pathway activation could be one mechanism of the niraparib-mediated immunostimulatory effects, suggesting that the integrity of the STING pathway may impact the potential for niraparib-induced immune activation. Therefore, factors regulating the integrity of the STING pathway, such as the DNA exonuclease Trex1, which is capable of degrading cytoplasmic DNA, resulting in attenuated activation of the STING pathway and IFN signaling, may impact the combination benefit in certain patient populations^49^. Exploring the role of STING and factors regulating STING and IFN signaling may reveal potential patient selection biomarkers for this combination.

PD-L1 induction on tumor cells by PARP inhibition has been previously reported as a mechanism underlying the combination benefit between PARP inhibitor and anti-PD-1 therapy^18^. In this study, the induction of PD-L1 expression was observed in only two tumor models (SK6005 and SA9003) (data not shown) but not in the rest of the tumor models examined, suggesting that the elevation of PD-L1 expression by PARP inhibitors could be context-dependent. For example, other ligands of the PD-1 receptor may contribute to immune checkpoint activity, which could potentially be overcome by anti-PD-1 therapies. In clinical settings, PD-L1 expression is not necessary for sensitivity to anti-PD-1 therapies, as a subgroup of PD-L1-negative patients also responded to anti-PD-1 therapies^50–52^. The lack of PD-L1 induction upon niraparib treatment in our study and in anti-PD-1-responding patients may also be attributed to insufficient PD-L1 detection sensitivity or transient PD-L1 induction. In contrast, it has been reported that tumor mutational load, microsatellite instability, and an immunogenetic tumor microenvironment may be better biomarkers for the anti-PD-1 therapy response, regardless of PD-L1 expression status^50,51,53^.

Combinations of PARP inhibition with anti-PD-1 therapies are currently being evaluated in clinical studies, and the results reported here support this therapeutic strategy. A critical next step is to understand the patient populations that will benefit most from this combination. *BRCA* mutational status was reported to be associated with higher predicted neoantigens, TIL infiltration, and PD-L1 status in ovarian cancers^54^, suggesting that tumors with *BRCA1/2* mutation or homologous recombination deficiency are more likely to benefit from PARP inhibitor and immune checkpoint inhibition. An unmet medical need remains in the homologous recombination-proficient populations, so it is important to evaluate whether PARP inhibitors can sensitize these tumors to immune checkpoint inhibition in clinical settings. Our results demonstrated a combination benefit in 6 of the 11 *BRCA*-proficient tumor models tested (data not shown), providing preclinical evidence supporting the clinical development of this combination. Recently, the combination benefit of PARP inhibition and anti-PD-L1 therapy was reported in the *BRCA* wild-type EMT6 breast cancer model^18^, consistent with our observation in *BRCA*-proficient models. Moreover, in the phase 2 TOPACIO trial, niraparib and anti-PD-1 combination therapy has demonstrated clinical benefit in a broad patient population including patients with the hard-to-treat *BRCA* wild-type platinum-resistant cancers and platinum-refractory ovarian cancers (2017 SGO Abstract#2990). Additional mechanistic studies and clinical tumor profiling will inform the future development of this combination in a broader patient population.

## Supplementary information

Supplemental figures
