# Supplementary material for: Niraparib activates interferon signaling and potentiates anti-PD-1 antibody efficacy in tumor models
Source: Sci Rep. 2019 Feb 12;9:1853. doi: 10.1038/s41598-019-38534-6 (PMC6372650; doi:10.1038/s41598-019-38534-6)
Supplement: Supplementary file 1 — Supplemental figures [file 41598_2019_38534_MOESM1_ESM.doc]

**Niraparib activates interferon signaling and potentiates anti-PD-1 antibody efficacy in tumor models**

Zebin Wang^1*^, Kaiming Sun^1*^, Yonghong Xiao^1*^, Bin Feng^1*^, Keith Mikule^1*^, XiaoYan Ma^2^, Ningping Feng^2^, Christopher P. Vellano^3^, Lorenzo Federico^3^, Joseph R. Marszalek^2,4^, Gordon B. Mills^3^, Jeffrey Hanke^1*^, Sridhar Ramaswamy^1*^, Jing Wang^1*#^

**Supplemental Figure 1**

**
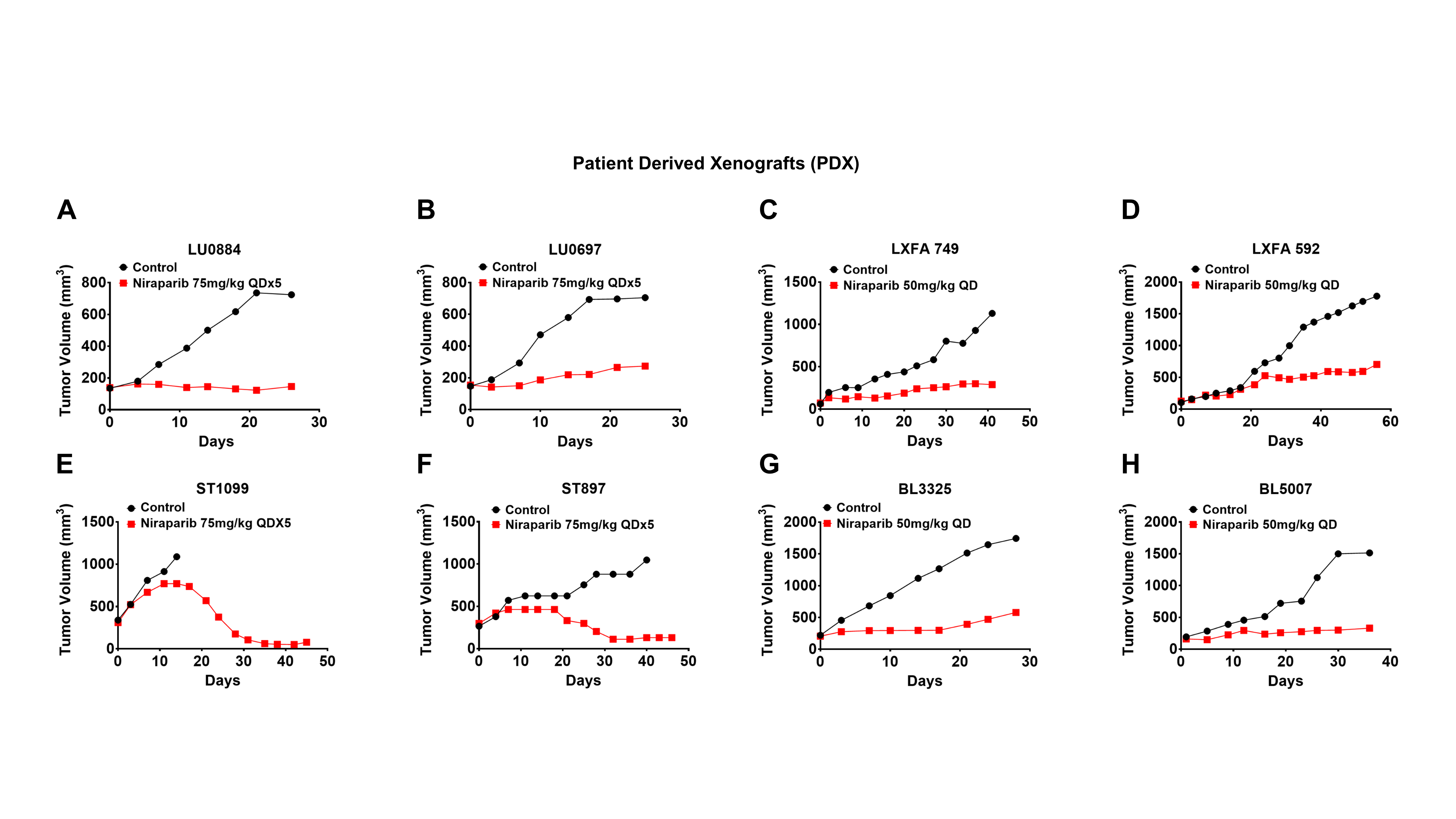
**

**Supplemental Figure 1:** Tumor growth in PDX models treated with control and niraparib. **(A-H)** Tumor growth in PDX models representing lung (LU0884, LU0697, LXFA749 and LXFA592), breast (ST1099, ST897) and bladder (BL3325, BL5007) cancers treated with control and niraparib.

**Supplemental Figure 2**


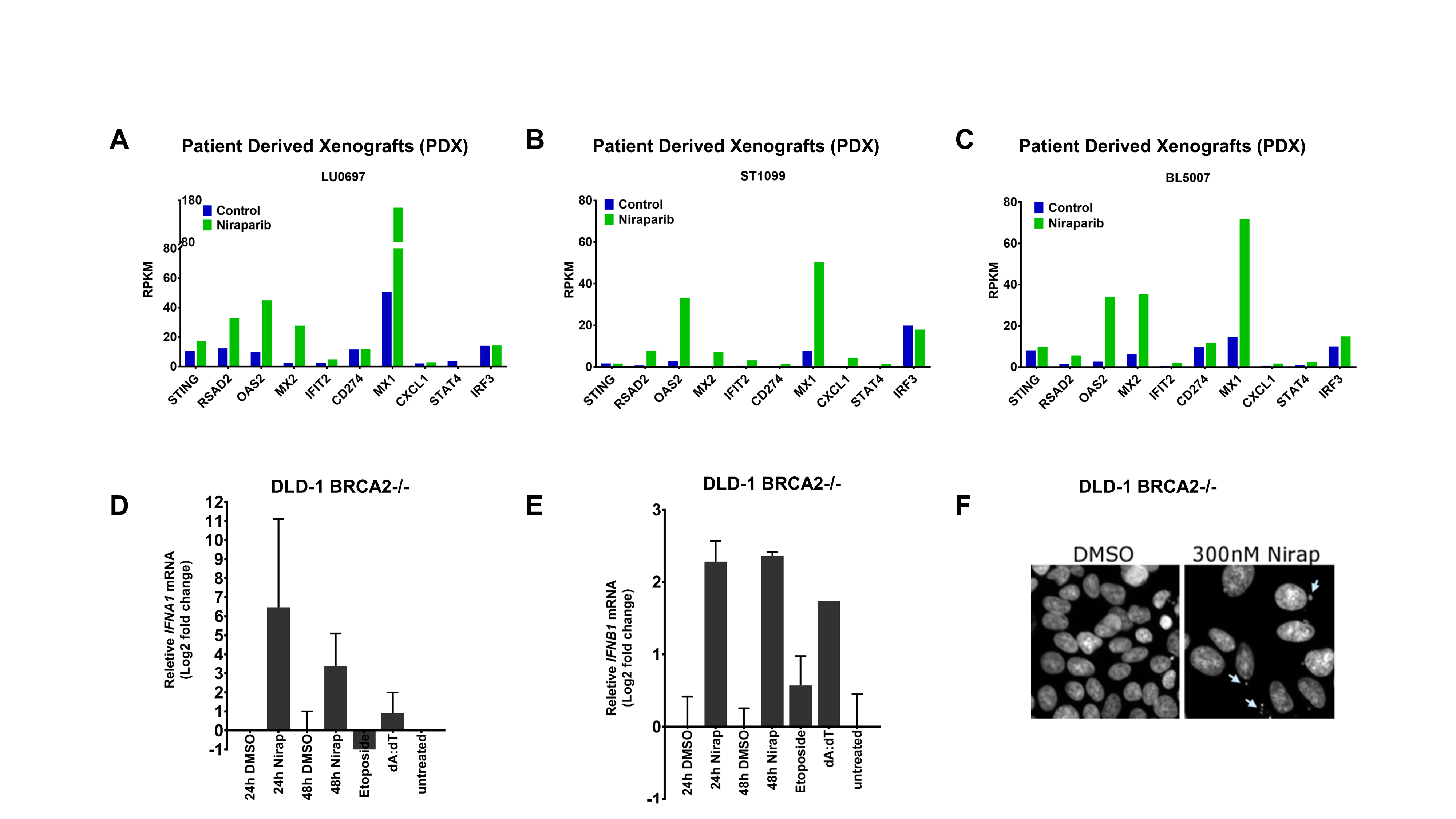


**Supplemental Figure 2**: Niraparib induced the expression of type I interferons and interferon-stimulated genes and promoted the formation of micronuclei.

(**A-C**) The expression of representative type I interferon-stimulated genes in tumors isolated in vivo from the PDX models LU0697, ST1099 and BL5007. (**D**) Expression of IFNA1 mRNA upon niraparib treatment (300 nM, 24 h and 48 h), etoposide treatment (50 µM, 18 h), Ara-C treatment (10 µm, 16 h), or dA:dT transfection (0.5 µg/ml) in DLD1 BRCA2-/- cells in vitro. (**E**) The expression of IFNB1 mRNA upon niraparib treatment (300 nM, 24 h and 48 h), etoposide treatment (50 µM, 18 h), Ara-C treatment (10 µm, 16 h), or dA:dT transfection (0.5 µg/ml) in DLD1 BRCA2-/- cells in vitro. (**C**) DAPI staining of nuclear structures following DMSO or 300 nM niraparib treatment in DLD1 BRCA2-/- cells in vitro. The arrows show micronuclei formation in niraparib-treated cells in vitro.

**Supplemental Figure 3**

**
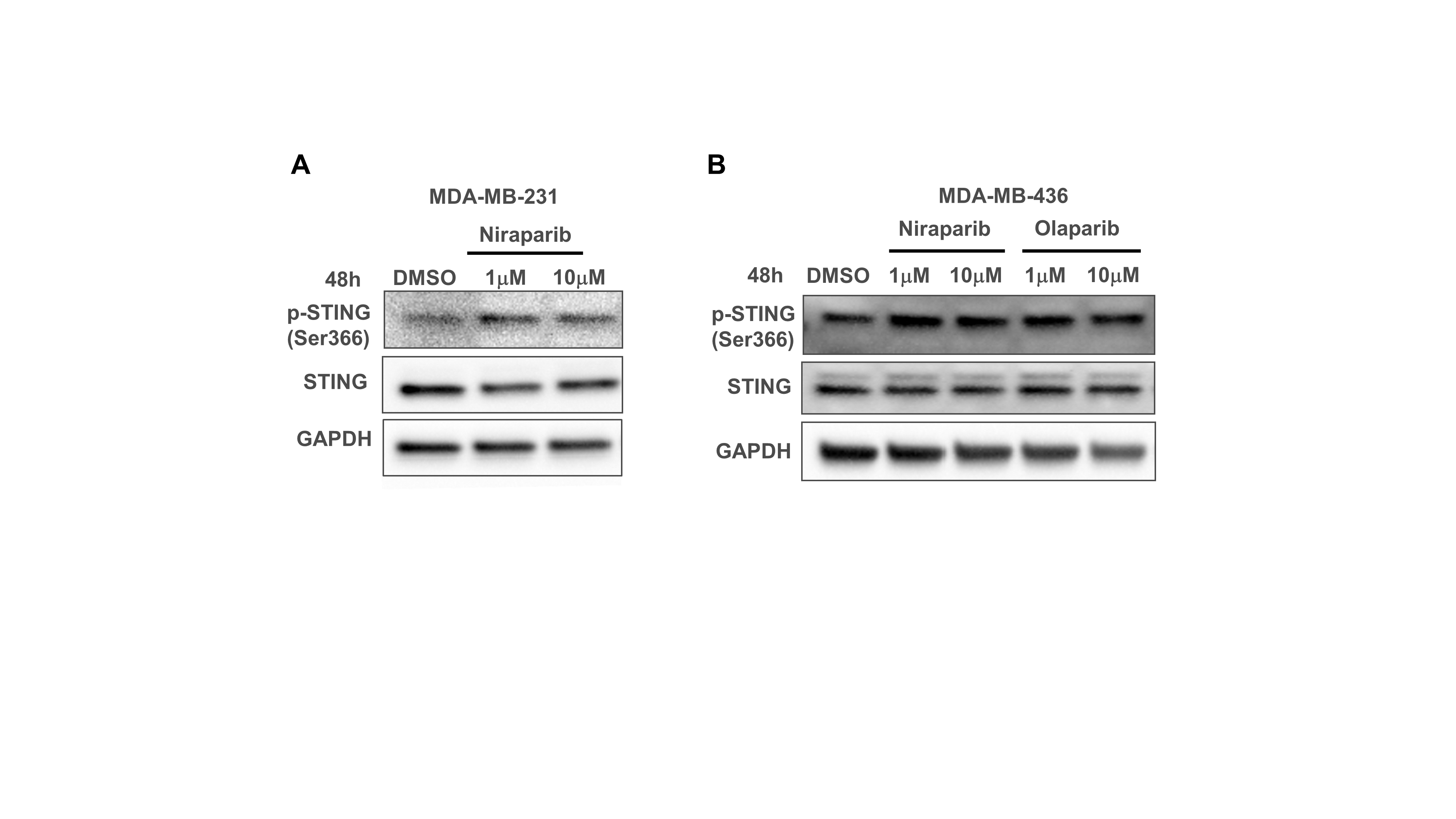
**

**Supplemental Figure 3**: PARP inhibitors induced p-STING (Ser366) expression.

**(A)** Protein expression of p-STING (Ser366), STING upon 1 µm and 10 µm niraparib treatment (48 h) by western blotting from the MDA-MB-231 lysate run on different gels indicated by the divider lines. **(B)** Protein expression of p-STING (Ser366), STING upon 1 µm and 10 µm niraparib or olaparib treatment (48 h) by western blotting from the MDA-MB-436 lysate run on different gels indicated by the divider lines.
